# How Disability Income Benefits Affect Employment for Persons with Disabilities in China: An Impairment-Based Work Disability Assessment Perspective

**DOI:** 10.3390/ijerph19063428

**Published:** 2022-03-14

**Authors:** Yuling Hao, Rikui Xiao

**Affiliations:** 1 Graduate Institute for Taiwan Studies, Xiamen University, No. 422, Siming South Road, Xiamen 361005, China; haoyuling@xmu.edu.cn; 2 Collaborative Innovation Center for Peaceful Development of Cross-Strait Relations, Xiamen University, No. 422, Siming South Road, Xiamen 361005, China; 3 China Disability Research Center, Xiamen University, No. 422, Siming South Road, Xiamen 361005, China; 4 Department of Sociology, Graduate Institute for Taiwan Studies, Xiamen University, No. 422, Siming South Road, Xiamen 361005, China

**Keywords:** disability income benefits, work disability assessment, employment

## Abstract

Reconciling the potentially conflicting goals between income benefits and employment has become one of the key issues in international disability policy for working-age persons with disabilities. Inspired by the often-criticized experience of disability income benefits having an exclusory effect on employment of persons with disabilities in welfare states by two mechanisms—the disincentive effect of the generous benefits based on the work incapacity of persons with disabilities and the impairment-based work disability assessment for attaining these benefits—this paper aims to examine how disability income benefits affect employment for persons with disabilities in China. Using the life-story interviews method, this paper found that the disability income benefits for persons with disabilities based on their work incapability create “quasi-employment” perceptions among recipients with disabilities. The impairment-based work disability assessment for attaining these benefits excludes persons with disabilities from formal employment but does open them to more informal employment. Implications for policy are discussed.

## 1. Introduction

Global disability policy for working-age persons with disabilities incorporates two potentially conflicting goals. One is to ensure that they participate in the labor market with pay as full and equal as possible; the other is to provide adequate and reasonable income benefits for those who cannot work because of disability [1,2,3]. Disability income benefits, as the earning replacement system based on work disability, are well known to be important for persons with disabilities to improve their standard of living [4,5]. However, it also has been criticized as a significant danger for marginalizing them from the labor market by two mechanisms. One is that the impairment-based work disability assessment for income benefits directly equates impairments with incapacity for work. Consequently, persons with disabilities have often been considered as naturally and unproblematically dependent on state benefits and automatically excluded from employment in modern welfare states [6,7,8]. Another is the generosity of income benefits being evidenced to encourage persons with disabilities to leave the labor market voluntarily [9,10]. Accordingly, disability policies have generally experienced a convergent shift from income maintenance to employment incentive over the last decades in welfare states [11,12].

In China, economy-centered development was the “central task” of national development over the last decades. One’s body had largely become the tool of production and competition in the labor-intensive economy. Consequently, persons with disabilities were increasingly excluded from the labor market. Meanwhile, a welfare state was emerging that served as a support system for the market economy [13,14]. Persons with disabilities have become the poorest of the poor. Aiming to improve their living conditions, they have been regarded as one of the key vulnerable groups entitled to basic provisions in the current national development plan [15]. Consequently, a series of redistributive policies have been developed in recent years. As Chinese national statistics showed, public transfer averagely accounted for 48.3% household income in households containing an adult with disabilities, as opposed to 18.3% of households in which no one was disabled in 2019 [16]. While public transfer benefits for persons with disabilities have become much better, their employment outcomes remain poor. In 2020, of the estimated 85 million persons with disabilities, approximately 8.6 million were officially registered workers. Among them, 49.2% were engaged in agriculture [17]. 

Some Chinese scholars have identified factors contributing to the poor situation of employment for persons with disabilities, mainly focusing on the mismatch between the supply and demand of jobs [18], the ineffectiveness of quota systems and anti-discrimination legislation [19], and the marketization ideologies [20]. However, while the medical definition of disability and the impairment approach to disability assessment are still prevalently used in disability policy in the context of a rapid expansion of redistributive benefits for persons with disabilities, little is known about how income compensation policy affects employment for persons with disabilities in China. Examining this issue is exactly what this paper aims to do. Our study showed that income benefits based on impairment-based work disability assessment have both empowering and debilitating effects on employment for persons with disabilities. This study can help those who are interested in China’s disability policy to better understand the current disability-related income benefits scenarios and the effects of both the work disability assessment and the income benefits on employment for persons with disabilities. 

The remainder of this paper is organized as follows: in the next section, we examine the theoretical issues related to work disability assessment for disability income benefits. Section 3 identifies the disability income benefits program and its basis for work disability assessment in China. The empirical evidence on the effects of disability income benefits on employment for persons with disabilities in China is reported in Section 4, followed by an integrated discussion of these results. 

## 2. Main Approaches to Work Disability Assessment for Disability Income Benefits

Disability income benefits aim at compensating the loss of earnings caused by work disability [21,22,23]. There is a broad consensus in the international literature that some basic approaches to work disability assessment have been developed historically [24,25,26].

The oldest strategy for work disability assessment is the impairment approach. This approach translates a given bodily impairment into a corresponding percentage of work incapacity. Thus, the severity of work disability is directly assessed from the description of an individual’s medical condition in connection with impairment [24,25]. Using an impairment approach to assess work disability is considered feasible and simple, with the important reason that medical professionals make good “gatekeepers” of public benefits [8]. However, an impairment approach is strongly criticized due to the inadequate and logically flawed use of impairment as a proxy for an individual’s capacity to work. Then, the functional limitation approach, which arose out of the response to criticisms against the impairment strategy, has been gradually accepted. This approach argued that work disability should be assessed by some basic activities or functional capacities as the essential predictors of work capacity. Therefore, a variety of Functional Capacity Evaluations worldwide were developed for and applied to work disability assessment [22,24]. 

While the two strategies mentioned above are still widely applied, another approach to work disability assessment termed the “disability approach” based on the insights of International Classification of Functioning, Disability and Health (ICF) has been gradually accepted. The ICF understands disability as a biopsychosocial phenomenon [27], and this understanding was adopted by the Convention on the Rights of Persons with Disabilities (CRPD) to conceptualize disability in 2006. Since then, ICF has been increasingly encouraged to conceptualize work disability in order to better understand, prevent, and manage it [28,29]. The ICF suggests disability is the interactional outcome of medical, functional, environmental, and personal factors. This disability approach considers the four determinants of work disability equally, rather than just focusing on impairment or functional limitation assessment [30].

Ideally, it is best to assess work disability for income compensation benefits on the basis of the ICF, which can directly assess the work capacity. However, while all countries around the world are confronted with addressing the complexities of disability determination for income compensation benefits, each country or transnational body has formed its own unique administrative characteristics and conceptual agreement of work disability assessment for income compensation programs. For instance, the European Union has been making efforts to develop the ICF-based disability assessment for income compensation programs among its member countries [26,31]. On the basis of the case studies of nine developed countries, Gerger et al. (2018) have identified three models for directly assessing work capacity for income compensation benefits—structured assessment, demonstrated assessment, and expert assessment—while the precise form of each model varies in different countries [30].

In China, disability is still defined and assessed in medical discourse. Our aim is to examine how disability income benefits based on impairment-based work disability assessment impact employment of persons with disabilities in China. Therefore, we identify China’s disability income benefits program and describe its approach to work disability assessment systematically in the next section.

## 3. Disability Income Benefits Based on Impairment-Based Work Disability Assessment in China

### 3.1. Dibao as a Disability Income Benefits Program

As discussed above, disability income benefits aim at compensating the loss of earned income due to work disability. An independent disability income benefits program has not been created for all working-age persons with disabilities in China’s disability policy. Aiming to reduce poverty among persons with disabilities, the “13th Five-Year Plan (2016–2020) for Accelerating the Process of Building a Moderately Prosperous Society for Persons with Disabilities” (13th Five-Year Plan) put forward that an adult with severe disabilities was eligible for the Minimum Living Standard Guarantee Program (*dibao*) by the establishment of a new household (*dandu lihu*) [32]. As the central program in China’s social assistance system, *dibao* targets households and provides cash transfers on the basis of the gap between *dibao* threshold and a household’s per capita income. Whether a person can obtain *dibao* eligibility highly depends on his or her household members’ income. However, the 13th Five-Year Plan has moved the entitlement of *dibao* for a person with severe disabilities away from household income to their severity of disability. 

According to the 13th Five-Year Plan, all provinces had developed provincial-level implementation measures for the entitlement of persons with disabilities to *dibao* eligibility on the basis of *dandu lihu* at the end of 2019. Reviewing these provisions, two common points of assessing work disability are identified as the eligibility criteria for *dibao*: (1) a person’s disability severity is assessed as degree-one or degree-two disability, both of which are equal to total work incapacity; (2) the person is unemployed. In other words, from the *dibao* standpoint, work disability refers to an inability to engage in any gainful activity resulting from degree-one or degree-two disability. *Dibao* benefits have translated into compensating the loss of earning-income caused by work disability. It is the only income benefits program for general working-age population of persons with disabilities in China.

### 3.2. The Impairment-Based Work Disability Assessment for Dibao

*Dibao* defines a person with degree-one or degree-two disability as having total work incapacity. In China, one’s severity of disability is stated in his/her Disability Identity Certificate as issued by the China Disabled Persons’ Federation. The severity of disability is determined by the nationally accepted medical definition of disability and disability assessment system. A Disability Identity Certificate is the basic certification for a person with disabilities’ access to various disability-based public resources. Thus, here, the disability assessment process is introduced to explain the impairment-based work disability assessment in *dibao*. 

A person with a disability is defined in the Law on the Protection of Persons with Disabilities, which was adopted in 1990 and last revised in 2008. This defines a person with a disability and the categories of persons with disabilities as: 

“One who has abnormalities or loss of a certain organ or function, psychologically or physiologically, or in anatomical structure and has lost wholly or in part the ability to perform an activity in the way considered normal.”

“Persons with disabilities include persons with visual, aural, speech, physical and intellectual disabilities, mental disorder, and multiple disabilities.”

The way in which to assess disability was not clearly defined in the Law on Protection of Persons with Disabilities. Hereafter, the disability assessment system has experienced three stages. In 1995, when the official national disability identification and registration system was established, a disability assessment standard for the First National Sample Survey on Disability in 1986 was adopted to identify persons with disabilities. In 2008, the Measures for the Administration of the Disability Identity Certificate of the *PRC* was issued by the China Disabled Persons’ Federation, which stated that the disability assessment standard for the Second National Sample Survey on Disability in 2006 was to be used to identity persons with disabilities. In 2011, the first National Standard for Disability Category and Degree (GB 26341-2010) was published. Six disability categories are classified according to the descriptions listed in the Law on Protection of Persons with Disabilities. Each type of disability severity is described as follows: degree-one disability (the most severe disability), degree-two disability (severe disability), degree-three disability (moderate disability), and degree-four disability (mild disability). The category of disability severity of individual is determined on the basis of the medical diagnosis by a local doctor designated for disability assessment in hospitals. In 2017, this standard was formally introduced to identify persons with disabilities by revising the Measures for Administration of the Disability Identity Certificate. 

In summary, China’s disability assessment approach is impairment-based. Both degree-one and degree-two disabilities as defined by *dibao* for persons with disabilities based on *dandu lihu* are determined in this system. As we have seen in the three approaches to work disability assessment used in income compensation policies around the world, the work disability assessment for *dibao* eligibility—equating degree-one or degree-two disability with the total loss of work capacity—completely marginalizes the functional limitations and environmental factors in the process of disability assessment. The World Disability Report revealed that, despite its significance, little was known about disability assessment or the variety of procedures used around the world [27]. On the basis of this knowledge gap, the World Bank issued a new report on disability assessment in the working-age population. However, there was still no detailed information about the disability assessment in China [22]. This paper provides a better knowledge of China’s disability assessment system and how it is used to assess work disability for attaining income compensation benefits. We move on now to examine how *dibao* eligibility based on *dandu lihu* affects employment for persons with disabilities in the next section. 

## 4. Data Sources and Method 

### 4.1. Data Sources

China’s *dibao* was first established in urban areas in the 1990s and extended to rural residents in 2007. Since the implementation of *dibao*, other social assistance programs have been gradually established. *Dibao*, as the central program of the social assistance system, has increasingly become the basis of eligibility for other programs. Chinese scholars have termed such a phenomenon as a “welfare bundle”, a serious problem in the *dibao* program [33]. For persons with disabilities, *dibao* is not only the eligibility criterion for other social assistance programs, but also for specific disability policies and social insurance programs. 

While the policy design frameworks of *dibao* and other policies associated with *dibao* eligibility are similar nationwide, disparities in benefits exist among municipal administrative districts. For practical reasons, we conducted our investigation in Xiamen City where the authors lived. We first mapped the benefits scenarios for persons with severe disabilities covered by *dibao* and other programs associated with *dibao* eligibility based on the *dandu lihu* (hereafter referred to as “the *dibao*-eligibility benefits”). Then, we selected 40 such *dibao* recipients to collect data for analyzing how income compensation policy affects employment of persons with disabilities. 

According to documents collected from the Xiamen Disabled Persons’ Federation, in 2020, a person with a severe disability could receive at least RMB 1700 per month from “the *dibao*-eligibility benefits”. In the same year, the minimum social wage was RMB 1800 per month, and the per capita disposable income was RMB 4845 per month in Xiamen City. Details about the disparities in benefits between *dibao* recipients with disabilities and persons with disabilities who did not receive *dibao* in Xiamen City are shown in Table 1. 

### 4.2. Method

#### 4.2.1. Sample

The sample consists of 40 individuals with severe disabilities who were entitled to *dibao* on the basis of *dandu lihu* in Xiamen City. Aiming to collect more accurate data of their own perceptions, we limited sampling to those who could express their thoughts clearly. In China, each community employs one Community Liaison Officer for persons with disabilities in charge of communicating and coordinating with the government, persons with disabilities, and their families with disability-related affairs in the Disabled Persons’ Federation system. The call for participation in the study was sent to these Community Liaison Officers through the Xiamen Disabled Persons’ Federation. They informed persons with disabilities who were receiving *dibao* and associated benefits on the basis of *dandu lihu* in their own communities. Finally, 40 individuals volunteered to participate in our study, and all of them had their full autonomy. The participants’ average age was 34.13 years (ranging from 20 to 51 years), and the sample included 24 men and 16 women; 55% had a physical disability, another 38% a visional disability, and 0.7% reported a mental illness. Regarding their educational background, 2.5% were illiterate, 20% had received primary school education, 22.5% middle school education, 40% high school education, 12.5% had a bachelor’s degree, and 2.5% a master degree. The participants’ education level on average was much higher than the average level of overall persons with disabilities in China. In terms of their work experience, 26 participants had work experience and 14 participants had no work experience. The relatively high education level and work experience may have contributed to their voluntary participation in our study. In addition, the sample only included people with a visional disability, a physical disability, or a mental disability. This may be attributed to their relatively low expression difficulties. Table 2 provides an overview of the demographic characteristics of the sample.

#### 4.2.2. Procedure

We used life-story interviews to answer the research question, as these enable researchers to peek into the inner world of their interviewees [34]. Participants were invited to tell their stories about how *dibao* based on their incapacity of work affected their employment. The researchers only intervened when the participants stopped talking about this topic and used prompts to extend their narratives aiming to obtain more information. The length of the life-story interviews ranged from 60 to 120 min. On average, interviews lasted 80 min. Most interviews involved the participant and the two authors of this paper. Interviews took place at the interviewee’s place of residence. The process of the data collection was completed once the saturation point was reached [35]. 

Ethical issues including privacy, content and confidentiality were considered throughout the study. It was voluntary for the interviewees to participate in the research. They were informed that they could leave the interview whenever they wanted. All participants recognized the authors’ scholarly identity and gave consent for their stories to be used for research. Each participant has been given pseudonyms and no personal details were exposed for tracing back to any of them. All quotations were originally in Chinese and translated by the authors. 

#### 4.2.3. Analysis 

Thematic analysis was used to analyze the data, following an inductive approach of open coding. Some codes were identified from public scholarship, whereas others emerged from the data. Aiming to ensure a reliable analysis, the interview data were analyzed by both authors of this article in order to reach the stage that Kvale (1994) calls “dialogic inter-subjectivity” [36]. We coded the data using the following steps. First, preliminary analysis was conducted upon finishing each interview, and constant comparisons were made between the new interview transcripts and the formerly coded data in order to update and revise the codes. Second, we combined and split these codes in order to identify the final categories and subcategories as presented in the finding section. This process was approached according to the recommendation of Lincoln and Guba (1985): identifying and establishing the existing codes (filling-in), querying in a different way (extending), inquiring into new or potential connections (bridging), and identifying new categories (surfacing). In the last step, we organized the categories together to represent the different dimensions involved in the effects of *dibao* on employment of persons with disabilities [37]. 

## 5. Research Findings

In welfare states, the disability income benefits have been criticized for producing employment disincentives arising from the generous disability income benefits on the basis of the work incapacity of persons with disabilities, as well as producing the exclusionary effects caused by the impairment-based work disability assessment for attaining these benefits. Thus, we present our research findings in two aspects: the effects of *dibao* benefits on employment of persons with disabilities, and the effects of impairment-based work disability assessment on employment of persons with disabilities in China. 

### 5.1. The Effects of Dibao Benefits on Employment of Persons with Disabilities

From our data, “the dibao-eligibility benefits” allowed the recipients with disabilities to create the “employed” perception shared by several participants. We refer to this perception as the “quasi-employment” perception. Specifically, five participants perceived that these benefits enabled them to play the roles as breadwinner, three participants perceived they were like the civil servants, and four participants argued that “the dibao-eligibility benefits” had transformed them into quasi-employers.

#### 5.1.1. Quasi-Breadwinner Role

For many participants, “the *dibao*-eligibility benefits” are the main or only source of household income among the family members. As indicated by a participant with a degree-one visual disability: “*Although I am not working, my wife and my son are all living on dibao and the associated benefits based on my disability. I*’*m still the breadwinner of the family*” (Participant 20). 

Remarkably, the data show that having “the *dibao*-eligibility benefits” as the stable income source has improved some participants’ marriages. A typical example is a participant with a degree-two physical disability. He once lived with his parents. Although they were a low-income household, they were not poor enough to be covered by *dibao*. Luckily, *dandu lihu* based on his severe disabilities allowed him to be eligible for “the *dibao*-eligibility benefits”. Soon afterwards, he got married. He proudly stated the reason: “*My wife has mild physical disabilities. In fact, we had the intention of establishing a family before my dibao eligibility. However, the main concern of her parents was my lack of stable income.* ‘The *dibao*-eligibility benefits’ made it possible for me to get married *two years ago when I was already aged 35. My parents-in-law believed the government could pay me a stable* ‘*salary*’ *due to my disability*” (Participant 39). 

#### 5.1.2. Quasi-Civil-Servant Status

Another category of participants even perceived they were like the civil servants as they received “the *dibao*-eligibility benefits” from the government on the basis of their disability. The quasi-civil-servant status was described mainly in three forms. 

One participant with a degree-one visual disability said “The government pays the premiums of resident old-age insurance and medical insurance for me, and I am fully reimbursed for the inpatient care expenses. These are all similar to the work welfare entitled to civil servants” (Participant 22). 

Another participant regarded herself as a person who was “*chi gongliang*”. *Chi gongliang*, a term popular in the planned-economy periods, referred to people whose wages were entirely paid by the government, and they could enjoy many welfare provisions, including housing, education, medical care, and child care. She explained that “*Due to dibao eligibility, my family members have benefited from social assistance for housing and medical care, and children*’ *education. The apartment we are living in now is public rental housing. I usually say that I am privileged to* ‘*chigongliang*’” (Participant 15). 

In addition, some participants expressed their quasi-civil-servant status from the ritual perspective, as illustrated by the following quotation from a participant with a degree-two physical disability: “*Each Spring Festival Day (Chinese New Year), local government leaders come to visit me; it appears that I am a retired civil servant*” (Participant 17). 

#### 5.1.3. Quasi-Employer Position

Besides the perception of quasi-breadwinner role and quasi-civil-servant status, a group of participants argued that “the *dibao*-eligibility benefits” have improved their self-perception and self-esteem by transforming them into employers mainly in two ways. 

Some participants indicated that the cash from “the *dibao*-eligibility benefits” allowed them to pay for care provided by their family members or others, which greatly reduced their shame and guilt. This phenomenon sounds like the policy practice in European countries that uses cash payments to transform persons with disabilities from passive service recipients to employers of personal assistants [6]. For example, a participant was paralyzed when she was 15 years old (10 years ago). She was mainly cared for by her mother. She stated, “*Even though she is my mother, caring for me had exhausted her physically and mentally. As she became gradually stressed and easily irritated, I tried to reduce the demands of care as much as possible. However, since I was entitled to dibao and associated benefits, I naturally have become confident to ask her for necessary assistance which I had tried my best to avoid before. The cash from the government allows me to feel I pay for her care to some extent, which reduces my shame and guilty while asking for assistance*” (Participant 21). Although categorizing disabilities in social policy has been criticized for creating stigma and dependence, people may not always perceive themselves like this [38,39]. Our interview data support this argument as well. Some participants viewed “the *dibao*-eligibility benefits” as enabling them to be self-employed on the basis of their disabilities. This meant that since their disability category in d*andu lihu* policy allowed them to receive “the *dibao*-eligibility benefits”, their disabilities were their assets rather than weaknesses. As one participant with a degree-two visual disability explained: “*We have been recognized as* ‘*valuable people*’ *rather than* ‘*disposable people*’ *by the state. Disability is my instrument to earn money from the government. I am employed by myself*” (Participant 10). 

To sum up, participants adopted a proactive attitude and analyzed how these benefits empowered them to create the quasi-employment perceptions, including their self-perceived role as breadwinner, civil servant, and employer. These benefits not only secured a basic standard of living for the participants and their families, but also improved their self-perception and self-esteem. 

### 5.2. The Effects of Impairment-Based Work Disability Assessment on Employment of Persons with Disabilities

*Dibao* eligibilities for persons with severe disabilities based on *dandu lihu* are underpinned by a total work incapacity. Accordingly, such kinds of *dibao* recipients are prohibited from engaging in formal employment. In this subsection, we not only describe how the participants were excluded from formal employment, but also report how they were included in informal employment. 

#### 5.2.1. Exclusion from Formal Employment 

Although persons with disabilities have been recognized as equal members in the society, they are marginalized from entering formal employment in institutional frameworks. There are two representative pieces of evidence. First, the recruitment processes for formal jobs public sector directly exclude most persons with disabilities by a mandated health examination according to the official standard termed “The General Standard for Civil Service Recruitment Health Examination (Trial, 2005)”. If the candidates fail to meet this health standard, they are assessed as lacking the capacity to work in the public sector. Second, China established a quota system in the early 1990s. As the most important tool for including persons with disabilities into formal employment in China, it has proven to be of very limited effectiveness [19,40] as has been observed worldwide [41]. 

Specific to persons with severe disabilities, the formal provisions on compensation and employment are incompatible. On the one hand, *dibao* recipients based on *dandu lihu* were prohibited from entering formal employment due to their legal incapacity to work; on the other hand, however, these people are encouraged to enter the labor market with the protection of the quota system. Therefore, persons with severe disabilities are limited to being compensated by *dibao* eligibility or employed with the protection of the quota system. Our data reveal that it was not the generosity of income compensation benefits but the poor accessibilities intertwined with discriminatory work environment that largely hampered persons with disabilities’ access to the quota system. 

In China, the environments of poor accessibility has raised a public discussion in the influential social media: there are about 85 million persons with disabilities—why are they invisible in public places? [42]. In our study, there was a typical participant who fell into such a dilemma. He had worked in a private enterprise as a mechanist before his paralysis caused by an accident. After acquiring the disability, his employer intended to retain his job through the quota system. However, physical barriers in his house, on public transportation, and at his workplace deprived him of this work opportunity. As he explained with disappointment: “*I am eager to return to my work rather than receive dibao and the associated benefits. However, the physical barriers imprison me at home……*” (Participant 22). 

Theoretically speaking, the quota system is the most important instrument for providing high quality jobs for persons with disabilities. However, the majority of job opportunities created by the quota system are low-quality, mainly resulting from both the employers’ practice and the low workability of persons with disabilities [19,40]. In our study, there was a participant with a degree-two intellectual disability who once was entitled to *dibao*. However, his mother asked him to leave *dibao* and work in a factory as a porter protected by the quota system. His mother made the decision for him to exit *dibao* with the moral reason that young people should work and enter the society, rather than live on government benefits and exit from the society. Unfortunately, after working for two years, he returned home and received *dibao* again. The primary reason for his resignation was not the income, but the discriminatory work environment, as highlighted by him: “*I had to do very heavy manual work. More importantly, the colleagues often discriminated against me. I felt so isolated and alone there……*” (Participant 17). 

In summary, equating impairments with work disability is still a mainstream thought that has been translated into the structuring of the policy system in China: focusing on disability benefits, rather than disability employment. As shown by these two typical participants’ experiences, they attempted to work rather than received “the *dibao*-eligibility benefits”, but even with the protection of the quota system, they still failed. This phenomenon largely resembles the criticisms against disability benefits based on the impairment-based work disability assessment in welfare states, that “they purchase the absence of ‘others’” [43]. 

#### 5.2.2. Inclusion in Informal Employment 

In contrast to the legally defined work incapacity based on an individual’s impairment, our participants had demonstrated actual work capacity in an informal employment, particularly in the digital economy. The digital economy emerged in the 1990s in China. However, it has risen from 10.0% to 38.6% in GDP [44]. Among our participants, 23 people with disabilities had work experience in the informal section. While three main forms of digital work performed by Chinese persons with disabilities have been identified—physical labor, social relations, and knowledge [20]—14 out of our 40 participants mainly engaged in two categories of digital work—physical labor and social relations. 

Physical-labor digital work is characterized as requiring low entry-level skills and flexibility, but it is time-consuming and physically demanding [20]. In our study, nine participants had experienced one or more of these jobs. In general, they viewed these jobs as enabling them to participate in employment using the unimpaired parts of their body and greatly increased their incomes. As one participant mentioned, “*I took some part-time work on the internet, mainly focusing on data entry for one company. Initially, they didn’t know I was paralyzed. About six months later, I told them my body situation and expressed the importance of this job for me. Unexpectedly, they increased the payment due to the quality of my work and a little bit of sympathy. The part-time earnings plus the dibao-eligibility benefits make my income the highest among my family members. Dibao eligibility has little impact on my employment*” (Participant 25). 

In respect to social-relation digital work, Qu (2020) mainly focuses on how persons with disabilities sell products on internet platforms with the help of their families, relatives, friends, and acquaintances [20]. Among our participants, five of them had such work experience. They viewed their work capacity as having changed situationally. As one participant explained, “*It is impossible for me to participate in employment the same as other able-bodied persons. My severely impaired body restricts me to stay at home. However, I could perform online sales. From this point, it is not accurate to say I have work incapacity. Anyway, thankfully dibao doesn’t prohibit me from engaging in e-commerce*” (Participant 13). 

Intriguingly, one participant with poor vison extended the meaning of social relations. She viewed selling goods to her circle of acquaintance, by nature, did not challenge the stereotype of a less-worthy persons with disabilities. Alternatively, she took her disabilities as giving her an advantage in conducting livestream e-commerce. Making her bodily impairment visible represented her spirit of self-reliance and personal strengthen and could help her attract more customers. She told us, “*I am good at singing. This merit and my disability enable me to attract customers and sell products for other distributors. I earn money in the form of commission. Moving my social relations away from acquaintances to my followers greatly improves my income and self-confidence. It’s amazing!*” (Participant 2). 

Both Participant 13 and Participant 2 took disabilities as their merits to perform digital work against the background of society’s attitudes towards persons with disabilities in China. Namely, they are expected to have self-esteem, self-confidence, self-reliance, and personal strength. Such discourse has not only been written in laws and documents, but also propagandized by constructing a “disability model” with the personal quality of “impaired in body but firm in spirit”. Thus, people are always inclined to support the self-reliant behaviors of persons with disabilities.

In summary, the persons with disabilities interviewed reflected on their capacity for work in the digital economy. They recognized that *dibao* eligibility based on *dandu lihu* had no negative impacts on their digital work. Similar to the international trend, the digital economy has provided more opportunities for persons with disabilities in the workforce [45]. Persons with severe disabilities who were defined as incapable of work have reconceptualized their work capacities in digital work. 

## 6. Conclusions and Discussion 

We aimed to examine how disability income benefits based on impairment-based work disability assessment affect the employment of persons with disabilities in China. Our results highlight the empowering and debilitating effects of income compensation policy on employment of persons with disabilities. 

In terms of the effects of disability income benefits on employment of persons with disabilities, we found that the “*dibao*-eligibility benefits” created a quasi-employment perception among our study participants. They perceived these benefits as enabling them to play the roles as breadwinner, civil servant, and employer. This overall picture suggests that “the *dibao*-eligibility benefits” have provided for their well-being in lieu of employment and greatly improved the standard of living of persons with severe disabilities in China. 

With regard to how impairment-based disability assessment for disability income benefits affects employment of persons with disabilities, the findings indicated that impairment-based work disability assessment for *dibao* eligibility excluded persons with disabilities from formal employment, but included them in informal employment, particularly in the digital economy. 

In the formal employment sector, *dibao* eligibility, based on compensating work incapacity, and the quota system, based on compensating those persons with a low capacity for work, have been conceptualized as competing choices for persons with severe disabilities. We found no evidence that the generosity of benefits from *dibao* eligibility encouraged persons with disabilities to leave the labor market as related studies have suggested in welfare states [9,10].This may be largely due to the fact that benefits remain low despite the number of benefit sources and very limited prospects in the labor market resulting from inadequate building accessibility, as well as institutional and attitudinal employment exclusion based on the ideology of “a medically impaired body, an equal but to-be-cared-for body, and a less capable body to be compensated for with either social support or individual endeavors” [46]. Like the previous studies [47], we restate that the current Chinese disability policy structure—focusing on developing social support, especially cash benefits based on the legislative category of disability—has recognized the right of persons with disabilities. However, we argue that the impairment-based disability assessment for these benefits contributes to the idea of treating persons with disabilities as passive recipients of care, rather than participators in formal employment in an accessible environment. Consequently, the policies aiming to reduce structural barriers to accessing formal employment remain underdeveloped. Welfare systems in Western countries also experience this and are criticized for the fact that passive benefits exclude employment of persons with disabilities [6,7,8]. In sharp contrast to the formal employment sector, another important finding of our study was that persons with severe disabilities were active in the digital economy sector. They even regard their impairments as instruments and assets in digital work, rather than barriers to formal employment. This is a forceful demonstration that it is time to rethink the appropriateness of impairment-based disability assessment in China. 

To conclude, *dibao* eligibility based on *dandu lihu* follows the logic that severe disability is equal with total work incapacity, and therefore *dibao* recipients should be automatically eligible for several other different-function programs. Although such an approach is more feasible and simpler for administration while helping persons with disabilities to receive incremental benefits, the following negative potentialities should be noted. 

First, attaching other benefit eligibilities to *dibao* eligibility has created new welfare disparities among persons with disabilities. In light of the complexity and multi-dimensions of disability, the definitions of disability and approaches to disability assessment must be differentiated according to the policy functions, at least including that for income maintenance, employment provisions, assistance in activities of daily living (ADLs), and equal opportunities [48]. *Dibao* eligibility based on *dandu lihu* serves as the income compensation benefits. Furthermore, the impairment-based work disability assessment for *dibao* per se grounds itself on an outdated assumption. Therefore, attaching other benefit eligibilities to *dibao* eligibility has resulted in a great mistargeting among disability policies. While there are no other specific studies, we show the benefit disparities between *dibao* recipients with disabilities and persons with disabilities who did not receive *dibao* in 2020 in Xiamen City in Table 1. *Dibao* eligibility and eligibility for other associated benefits based on an individual’s impairments may lead to “some are more equal than others” in social policy. 

Second, conceptualizing work and benefits as competing choices for persons with severe impairments ignores the environmental barriers that hamper their access to employment. As we reviewed in Section 2 of this paper, disability is an interactive product of health conditions and environmental factors. Thus, there has been a great attempt to assess work disability for income compensation benefits based on ICF insights with the purpose of incorporating the environmental dimension into the process of disability assessment. Impairment-based work disability assessment originated from an era when physical labor was dominant [8]. The labor market has witnessed tremendous evolutions and changes in the last decades in China. The large number of persons with disabilities engaging in the digital economy have fully demonstrated that it is not appropriate to exclude persons with impairment from formal employment on the basis of equating impairments with being “unable to work” directly [2]. Instead, there should be more policies aiming to address environmental barriers for impaired persons’ access to employment, or at least this should be given equal focus as the passive benefits. 

Third, policies for equal participation have been underemphasized. While “Equality, Participation, Sharing” has been established as the development ideology of China’s disability affairs [49], three kinds of disability policies—social protection, labor inclusion, and civil rights—should be developed in parallel. However, with public policy per se as the main source of constructing images of persons with disabilities as being dependent and incapacitated, *dibao* and other benefits associated with *dibao* eligibility based on total work incapacity have strengthened the image of persons with disabilities as being incapable. Consequently, the active force driving the policies designed to improve equal participation would be weak or even ignored, given what has been evidenced currently by the rapid expansion of benefits and poor employment outcomes for persons with disabilities. 

In a nutshell, our study found that income compensation benefits for persons with severe disabilities have evidently improved their well-being but not produced disincentives against employment for them. The impairment-based work disability assessment for *dibao* and related benefits eligibilities have created new welfare disparities among persons with disabilities and negatively impacted policies for promoting equal employment for them. Similar to the worldwide trend, public budgets, technical development, and rights protection for persons with disabilities have driven policy makers in China to reflect on the relationships among disability, welfare, and work. Meanwhile, they also should invest more effort in the development of strategies for moving away from a medical definition of disability and an impairment-based work disability assessment in disability policies, as has been discussed in recent years. 

There are several limitations of the current study. Considering the complexities of policies responding to disability, we only looked at the income compensation policies for working-age persons with disabilities who hold the Disability Identity Certificates. Secondly, since only *dibao* eligibility based on *dandu lihu* is characteristic of the income compensation function, the study participants were limited to persons with severe disabilities. In addition, considering the disparities in benefits offered throughout China and the highly limited statistics nationwide on *dibao* recipients with disabilities, our qualitative data were collected and analyzed only on the basis of Xiamen City. Care must be taken when applying these findings to other social contexts. Keeping these caveats in mind, it is also important to note that our smaller-scale qualitative research offers a great deal of information regarding how income compensation policy affects employment for persons with disabilities in China. This topic requires further intensive examination as China is shifting from a production-centered society to a people-centered society that greatly focuses on improving the wellbeing of everyone. 

## Figures and Tables

**Table 1:** Differences in benefits between *dibao* recipients with disabilities and people with disabilities who did not receive *dibao* in 2020.

Programs		*Dibao* Recipients with Disabilities		People with Disabilities Who Do Not Receive *Dibao*	
Social insurance	The Resident Basic Medical Insurance	(1) Government-funded full subsidies for personal contributions (RMB 320/year) (2) 100% of the benefit coverage reimbursement for inpatient care (RMB 100/year)		(1) No government-funded subsidy for contribution (2) 80% of the benefit coverage reimbursement for inpatient care	
	The Resident Basic Old-Age Insurance	Government-funded full subsidies for the personal contributions (RMB 100/year)		None	
Social assistance	*Dibao* Benefits	RMB 890/month		None	
	Housing Assistance	Priority is given to *dibao* recipients			
	Education Assistance				
	Medical Assistance				
Specific disability policy	Living Assistance for Poor Persons with Disabilities	Degree-one and degree-two disabilities (RMB 510/month)	Degree-three and degree-four disabilities (RMB 430/month)	Degree-one and degree-two disabilities (RMB 430/month)	Degree-three and degree-four disabilities (RMB 340/month)
	Care Subsidy for Persons with Severe Disabilities	RMB 360/month		None	
	Spring Festival (New Year) Allowance	RMB 1000 once a year		None	
Other benefits		Some temporary benefits for disadvantaged population		None	

Source: Materials from Xiamen Disabled Persons’ Federation.

**Table 2:** Demographic characteristics of the sample.

Participant	Gender	Age	Educational Level	Type of Disability	Marital Status	Work Experience
1	W	32	High school	Vision	Single	No
2	W	20	High school	Vision	Single	Yes
3	M	45	Primary school	Physical	Married	No
4	W	24	Middle school	Physical	Single	Yes
5	W	30	Primary school	Physical	Single	Yes
6	M	34	Middle school	Physical	Single	Yes
7	M	47	Primary school	Vision	Divorce	Yes
8	M	41	Middle school	Vision	Married	Yes
9	M	32	High school	Physical	Married	Yes
10	W	34	Primary school	Vision	Married	No
11	M	42	Bachelor	Physical	Married	Yes
12	M	26	High school	Vision	Single	Yes
13	W	24	High school	Physical	Single	Yes
14	M	30	High school	Physical	Single	Yes
15	W	40	Illiteracy	Physical	Married	No
16	W	34	High school	Physical	Married	No
17	W	30	Middle school	Physical	Married	No
18	M	32	Middle school	Vision	Single	No
19	M	33	Bachelor	Physical	Married	Yes
20	M	37	Primary school	Vision	Married	No
21	W	25	Middle school	Physical	Single	No
22	M	50	Primary school	Vision	Single	No
23	M	36	Master	Physical	Married	Yes
24	W	44	Primacy school	Vision	Married	No
25	M	26	High school	Physical	Single	Yes
26	M	29	Bachelor	Physical	Single	Yes
27	M	25	High school	Mental illness	Single	Yes
28	W	40	High school	Physical	Married	No
29	M	42	Bachelor	Mental illness	Divorce	Yes
30	M	51	Primacy school	Vision	Married	No
31	M	42	High school	Physical	Married	Yes
32	W	25	High school	Mental illness	Single	Yes
33	W	22	High school	Physical	Single	Yes
34	M	48	Middle school	Vision	Divorce	Yes
35	M	27	Bachelor	Physical	Single	Yes
36	W	36	High school	Vision	Married	Yes
37	M	32	High school	Vision	Married	Yes
38	W	26	High school	Vision	Single	Yes
39	M	37	Middle school	Physical	Married	No
40	M	35	Middle school	Physical	Single	Yes

## Data Availability

We did not report any data.
